# Participatory approach to design social accountability interventions to improve maternal health services: a case study from the Democratic Republic of the Congo

**DOI:** 10.1186/s41256-017-0024-0

**Published:** 2017-02-06

**Authors:** Eric M. Mafuta, Marjolein A. Dieleman, Leon Essink, Paul N. Khomba, François M. Zioko, Thérèse N. M. Mambu, Patrick K. Kayembe, Tjard de Cock Buning

**Affiliations:** 1 Kinshasa School of Public Health, Faculty of Medicine, University of Kinshasa, P.O. Box: 11850, Kinshasa I, Kinshasa, Democratic Republic of the Congo; 2 Athena Institute, Faculty of Life Sciences, VU University Amsterdam, Amsterdam, The Netherlands; 3 Royal Tropical Institute, Amsterdam, The Netherlands; 4 Cordaid Representative Office, Kinshasa, Democratic Republic of the Congo; 5 Medicus Mundi Representative office, Kinshasa, Democratic Republic of the Congo

**Keywords:** Interactive learning and action, Involving users, Facility delivery, Maternal mortality, Quality of care, Health service responsiveness, Dialogue Model, Social accountability, Voice, DR Congo

## Abstract

**Background:**

Social accountability (SA) comprises a set of mechanisms aiming to, on the one hand, enable users to raise their concerns about the health services provided to them (voice), and to hold health providers (HPs) accountable for actions and decisions related to the health service provision. On the other hand, they aim to facilitate HPs to take into account users’ needs and expectations in providing care. This article describes the development of a SA intervention that aims to improve health services responsiveness in two health zones in the Democratic Republic of the Congo.

**Methods:**

Beneficiaries including men, women, community health workers (CHWs), representatives of the health sector and local authorities were purposively selected and involved in an advisory process using the Dialogue Model in the two health zones: (1) Eight focus group discussions (FGDs) were organized separately during consultation aimed at sharing and discussing results from the situation analysis, and collecting suggestions for improvement, (2) Representatives of participants in previous FGDs were involved in dialogue meetings for prioritizing and integrating suggestions from FGDs, and (3) the integrated suggestions were discussed by research partners and set as intervention components. All the processes were audio-taped, transcribed and analysed using inductive content analysis.

**Results:**

Overall there were 121 participants involved in the process, 51 were female. They provided 48 suggestions. Their suggestions were integrated into six intervention components during dialogue meetings: (1) use CHWs and a health committee for collecting and transmitting community concerns about health services, (2) build the capacity of the community in terms of knowledge and information, (3) involve community leaders through dialogue meetings, (4) improve the attitude of HPs towards voice and the management of voice at health facility level, (5) involve the health service supervisors in community participation and; (6) use other existing interventions. These components were then articulated into three intervention components during programming to: create a formal voice system, introduce dialogue meetings improving enforceability and answerability, and enhance the health providers’ responsiveness.

**Conclusions:**

The use of the Dialogue Model, a participatory process, allowed beneficiaries to be involved with other community stakeholders having different perspectives and types of knowledge in an advisory process and to articulate their suggestions on a combination of SA intervention components, specific for the two health zones contexts.

**Electronic supplementary material:**

The online version of this article (doi:10.1186/s41256-017-0024-0) contains supplementary material, which is available to authorized users.

## Background

With a ratio of 846 maternal deaths per 100,000 live births [1], the Democratic Republic of the Congo (DRC) is one of the countries presenting with a high maternal mortality. Three-quarters of these deaths occurred during childbirth and postnatal periods [2]. Interventions to reduce maternal morbidity and mortality emphasize facility-based childbirth and skilled attendance during delivery with timely referral for emergency obstetric care if complications occur [3, 4]. Progress towards achieving a reduction of maternal deaths has been slowed because improvements require overcoming financial, geographical and socio-cultural barriers to accessing skilled birth attendants, as well as poor quality of care at facilities.

To address this situation, innovative strategies beyond providing skilled personnel, improving equipment, and infrastructures are needed [5, 6]. Some of these strategies have to deal with improving women’s service uptake, by improving quality of care and the health provider-user relationship. One of these strategies consists of the use of social accountability mechanisms. Social accountability mechanisms are mechanisms that lead health service providers to take into consideration users’ expectations and needs [7, 8]. They aim to improve the responsiveness and behaviour of health providers towards users [7, 8]. Social accountability relies on civic engagement, i.e. in which citizens and/or civil society organizations participate directly or indirectly, formally or informally in exacting accountability [9] and bringing politicians, policy makers and healthcare providers to account as responsible for their performance [10–13].

While a growing body of literature examines social accountability and describes its mechanisms [13–16], little is known on how to shape social accountability mechanisms to fit a specific context and how to involve beneficiaries in this process [13, 17].

According to Georges, poor involvement of beneficiaries in the design of most health programmes have limited their efficacy. As policy makers are becoming aware of this, increasingly beneficiaries are involved in decision-making regarding health policy, treatment and health research, mainly in high-income countries [18]. To develop a functional social accountability mechanism, a multi-phased participatory approach is useful, involving a broad range of actors with different perspectives and types of knowledge. An example is the Dialogue Model [19, 20] which includes a joint learning process among stakeholders [20].

A study on social accountability in maternal health in two health zones in the DRC that we conducted in 2013 showed that very few women voiced their concerns and complaints to health providers, although study respondents asserted the existence of inappropriate care in local health services. Interviews revealed that women in rural area are not used to expressing their concerns and they did not mention the quality of care or health providers’ behaviour. In addition, the study showed that women did not know how to transmit their concerns to relevant actors and decision makers or how their concerns were managed within the health services. This study also revealed that this situation is mainly due to the absence of procedures to express concerns, the lack of knowledge thereof, fear of reprisals or of being misunderstood by health providers as well as factors such as age-related power, ethnicity, and the low socio-economic status of women [21]. To develop interventions based on these outcomes some questions required answering in the light of these findings. Which social accountability mechanisms are needed in order to improve maternal health services responsiveness and performance? How could community groups be involved in designing these social accountability mechanisms to make them more relevant?

This article describes the development of a social accountability intervention that aims to improve maternal health services responsiveness and performance in two health zones in the DRC, by involving beneficiaries, representatives of the health sector and local authorities in the advisory participatory process using the Dialogue Model.

## Methods

### Study design

In order to answer the research questions, we developed a participatory action research process, based on the Dialogue Model [19, 20] in two health zones. The two health zones (HZs): Muanda HZ in Kongo Central Province in the southwest and Bolenge HZ in the Equateur Province in the northwest, were purposively selected according to the presence of a health partnership supporting or aiming to support an intervention containing a social accountability mechanism [21].

**Fig. 1:**
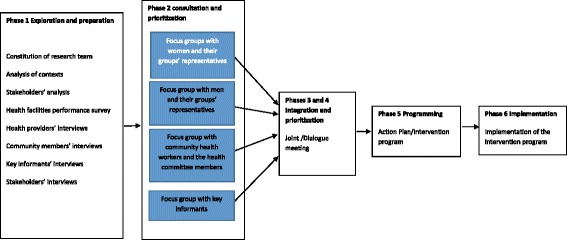
Visualization of the Dialogue Model process

The Dialogue Model was chosen as a participatory action approach as it was found suitable to be used when dealing with complex phenomena occurring in an interface, as it allows to achieve appropriate participation [18], and offers guidelines and principles on how to consult and integrate issues from different stakeholder groups in an advisory process. It is based on six principles: active engagement of beneficiaries, conducive social conditions, respect for experiential knowledge, mutual learning, emergent and flexible design, and facilitation process. It is roughly divided into six phases, the product of a phase serving as inputs for the following phase. The six phases are: initiation and preparation, consultation, prioritization, integration, programming, and implementation [22]. The Dialogue Model was slightly adapted for its application to the context of community participation in intervention development in the two health zones by putting two of the six phases together: integration and prioritization phases, and four of its phases were conducted during this reported process (Fig. 1). The implementation phase of the Dialogue Model (DM) was considered as mandate of the health partners and health providers and is beyond the scope of this paper.

First, the initiation and preparation phase was conducted. This included identification of organisations involved in maternal health, an invitation to the organisations which consented to participate in the project to attend a workshop on the project contents and approach and the establishment of a partnership between the research team and health sector partners. The inclusion criteria for the partnership was that the organisation is a DRC health partner that implements or plans to implement an intervention with a social accountability component and is interested in research on social accountability. In addition, a context analysis and an exploratory study were carried out, mapping contextual factors that influence social accountability initiatives and interviewing relevant actor groups about the existing social accountability mechanisms at the two research sites. Research methods and findings of the exploratory study are described elsewhere [21].

Second, the consultation phase was carried out to obtain the reactions of actor groups in the two health zones and of the health partners on the findings of the exploratory study and the context analysis carried out in phase 1, and develop lists of intervention suggestions from each actor group to improve social accountability for maternal health services. In this phase, at the national and provincial levels, meetings were organized with health sector partners involved in maternal health, including community representatives, ministry of health officers and non-governmental organizations representatives. The aim of these meetings was to share and discuss findings, and inform policy makers. Policy briefs were developed and disseminated. This step will be described elsewhere.

In the two health zones, four focus group discussions (FGDs) were held with four different actor groups: women beneficiaries and their community groups’ representatives (*n* = 12), men and their community groups’ representatives (*n* = 12), community health workers and health committee members (*n* = 12); representatives of the health sector at local level including health providers, health zone officers, health partners, and local authorities (*n* = 12). As it is equally important to have men involved in maternal health, they were also involved in equal numbers with women in the process. A FGD guide was used to structure the discussion. In each FGD, participants were invited to discuss the extent to which the results of the context analysis and the exploratory research reflected the reality of their community (See Additional file 1). Subsequently, they were asked to provide suggestions for improving the situation. These suggestions were summarized in a list of interventions components and validated by the participants. The research team also informed participants about the next phase: integration and prioritization.

The third phase combined integration and prioritization. In this phase suggestions coming from the four participating FGDs were integrated into one shared intervention. The integration and prioritization was organized as a dialogue meeting at each site. In this meeting representatives of all participating FGDs were convened to discuss suggestions and perspectives of the different groups and to integrate them in one intervention proposal.

From the list of people who had participated in previous phases of the project, twelve participants were invited to attend the dialogue meeting at each research site on the basis of their background, willingness to enter into a dialogue, open-mindedness, capability to express themselves clearly, succinctly and constructively as assessed during the focus groups and their availability. Participants in equal number for each group included beneficiaries: men and women (*n* = 4), community health workers and health committee members (*n* = 4), and representatives of the health sector and local authorities (*n* = 4). The research team provided assistance to the beneficiaries groups. Prior to the meeting, the research team discussed the suggestions of their own group with selected participants in order to prepare them for the integration meeting and provide training on negotiation and advisory skills, especially with community members. The integration meeting was held in a quiet place and at an appropriate time, facilitated by research team members using non-technical language. The construction of an integrated proposal was done by the participants in the meeting using a process of ordering and ranking: Firstly, the list of suggestions of each actor group was separately and repeatedly read by participants so as to become familiar and to identify the main ideas for improving social accountability. Then, they were invited to regroup suggestions having similar meanings using post-it, forming intervention components and to propose a description of the content of each intervention component. For each discussion, each participant group was allocated equal conversation time. Subsequently, using the same procedure, intervention components targeting similar actors were further regrouped into intervention main-components. The integration process was completed by describing each intervention of the main-components. At the end of the meeting, the main result was a single integrated intervention proposal, which was discussed and validated by the participants. The research team informed participants about the next step: the programming phase.

The fourth phase, programming, was conducted at a national level with the aim of developing a social accountability intervention for implementation. The two community intervention proposals were used as a basis for formulating social accountability interventions that are to be implemented in the two HZs. This phase was conducted during a workshop held in Kinshasa by the research team and the health partners, specifically representatives of Cordaid, Medicus Mundi and officers from the Ministry of Health. They discussed the results of the integration phase, and selected intervention components for social accountability. Considerations for the selection of suggestions from the integration phase to include as social accountability intervention components in programming phase comprise: (1) the technical feasibility to implement the suggestion taking into account the current health policy and; (2) the possibility to improve or to use existing health sector intervention or community elements. After that they discussed modification to be introduced in existing interventions where opportunities for social accountability exist such as in the performance based financing intervention in Muanda HZ and the community based health insurance intervention in the Bolenge HZ. The programming workshop was facilitated by the research team, and was audio-taped and transcribed verbatim.

### Participants in different phases

Participants in the FGDs during consultation phase and integration phase were sampled purposively in relation to relevant stakeholder groups: among community members (women, men, representatives of their community groups, community health workers and health committee members) and representatives of the health sector (health providers, health zone management officers, local health partners), and local authorities. Other inclusion criteria used were: (1) aged between 17 and 75 years, and (2) living in the community for more than 2 years. Based on the inclusion criteria, a list of people to invite was established with the collaboration of community health workers and local authorities. Participants were sampled using a systematic sampling procedure in order to select 12 persons for each category if their number was more than 12. In a category where participants were less or equal to 12, all were *de facto* included. These persons were contacted and invited to participate using community health workers. Those who expressed a willingness and interest to participate were included in the study. This allowed the researchers to capture the maximum information and experiences of the different stakeholders. Community members were approached outside their homes and health sector representatives and local authorities in their workplace, and invited to participate in the FGDs and integration meetings.

The programming workshop gathered research partners comprising of officers from the Ministry of Public Health, health partners (Cordaid and Medicus Mundi) and the research team.

### Data collection and documentation of the participatory process

Phases 2 to 5 were organized from February to May 2015 and were facilitated by the research team. The FGDs and integration meetings were held in a quiet place, far from other people to optimize privacy and lasted on average for approximately 2 h. They were conducted in Lingala and in French, audio-recorded with the consent of the participants. A brief report of each meeting was written by the research team members and orally discussed with the participants for member check.

A debriefing session among the research team was held after each group meeting during which themes, impressions of the findings and procedures were discussed and documented in field notes, and group meeting reports were written. The field team was supervised by three senior researchers.

### Data analysis

Recorded group meetings were generally transcribed *verbatim* in Lingala, translated into French and checked by two team members, then, combined with field notes, and mini-reports produced by the research team after each meeting. These transcripts were analysed using an inductive content approach in order to identify emergent themes and trends in the data [23]. The research team read and re-read the transcripts to become familiar with the whole data set. Subsequently, the analytical approach was to label participants’ suggestions and coded in sub-categories. Several sub-categories having similar ideas or relating to the same topic were used to construct categories. Categories in turn were regrouped into themes by grouping categories relating to similar actors. Throughout the analysis, the team used notes from the two integration meetings to describe categories and themes. Moreover, the use of the notes from the integration meetings helped to assure the trustworthiness of the analysis. The analysis process was then discussed with two supervisors (MD and TDCB).

## Results

Overall, 121 participants aged 22–67 were involved in the process. Women represented around one-third (*n* = 51). The level of education of participants ranged from no education to master’s degree. In Table 1 an overview is given of the characteristics of the participants in the FGDs, integration meetings and workshop.

**Table 1:** Characteristics of participants in group meetings

Participants	Location	Number	Sex		Age	Education	
			M	F		Lowest	Highest
Focus groups							
Representatives of the Health sector and local authorities	Muanda	12	9	3	30–65	P5	MPH
	Bolenge	8	7	1	31–45	U3	G/MD
Community Health workers and Health committee members	Muanda	12	6	6	23–67	P6	U3
	Bolenge	12	7	5	25–65	P4	U1
Men and men’s groups representatives	Muanda	12	12	-	25–57	P6	U2
	Bolenge	12	12	-	31–63	P4	U1
Women and women’s groups representatives	Muanda	12	-	12	23–45	P6	S6
	Bolenge	12	-	12	22–54	NE	S6
Dialogue meetings	Muanda	12	6	6	32–55	S2	MPH
	Bolenge	12	6	6	33–60	S3	G/MD
Health partners	Kinshasa	5	5	0	32–55	G	MPH
Total		121	70	51	22–67	No	MPH

*NE* No education, *P* Primary school, *S* Secondary school, *U* Undergraduate, *G* Graduate, *MD* Medical doctor *MPH* Master in Public Health, Lecture: *S2* second level of secondary school

### Consultation phase

Overall, four FGDs were organized in the consultation phase at each site. In general, combining data from both sites, participants from different backgrounds initially provided 48 suggestions which partly overlapped (Table 2). Participants of these FGDs made suggestions according to their knowledge and experiences of the local setting, trying to find solutions that they thought were important from their perspectives. Women, men, community health workers (CHWs) as well as local authorities at both sites suggested the use of CHWs as intermediary and interface for collecting the population’s needs and expectations and transmitting them for discussion at the health committee’s meeting. They suggested that the health committee evaluates health services at local level and creates a feedback loop to inform the population about the health committee’s decision using the same CHWs. Furthermore, both women and men suggested that the capacity of the community on maternal health matters developed by sensitization and awareness activities. Actions to improve health providers’ attitudes toward voice and the management of voice at facility level or the involvement of health sector supervisors such as the health zone management officers were also suggested by the community FGDs. The proposed actions included the training of health providers because participants thought that health providers needed to be sensitized to respect the voice and rights of patients. Almost all their suggestions were agreed upon by other groups.

**Table 2:** Suggestions for improving social accountability in maternal health services in local settings

Muanda	Bolenge
Key informants	
- To reach out to the population about expressing their concerns and complaints, and health providers about being responsive; - The awareness activities at population level would be done by CHWs mainly during home visits; - To provide CHWs with a small incentive; - To improve community recognition of CHWs through an election process in the community; - To train CHWs for improving their activities; - The health providers could also get population’s voice through community survey conducted in PBF settings; - to initiate periodic meetings between CHW, HC members, health providers and decision-makers to share and discuss health issues, - To encourage the participation of the HZMT Officer in these meetings; - To work on improving the women’s confidence in CHWs - To reduce the “cutting” practices sometimes used when writing Health committee’s meeting report.	- To use CHWs for reporting complaints and concerns about health services; - To reach out to the population about all existing social accountability mechanisms; - To improve the work of CHWs by an adequate trainings and their choice through community election; - The training of CHWs would be done by the HMT members in charge of community activities; - To improve the functioning of health committee; - To reach out to health providers for improving their responsiveness; - To document population’s complaints and concerns using a formal system of records; - To include local authorities and community leaders specifically religious leaders in the process; - To bring forward complaints and concerns about GRH using CHWs, who could report them during Health committee meeting and through this latter’s report, to HZMT officer. - To use mechanisms of Community Health Insurance
Community health workers and Health committee’s members	
- To still continue to receive from the population concerns, questions and complaints using home visits; - To bring them forward to health providers during dialogue meeting and the health committee meeting; - To make all decision as a group and not individually; - To sensitize population to report their concerns; - To ask HMTO to be present in their meeting in order to get complaints and concerns about the GRH; - To recognize that their number is not optimal given the sunk cost of working without being paid and the difficulty of enlisting local associations to become involved in non-remunerated activities.	- Observed that all accountability is centred on the nurse in- charge, who receives information from the health committee and has to be responsive with his team; - To organize two meetings, one for the CHWs and their delegates in the health committee and the health committee meeting; - To transmit decisions of the health committee to the CHWs for closing the loop; - To organize public meetings putting together the health committee, the health centre providers and the community with the possibility of public questions and answers. - To invite to these meetings local associations’ representatives and authorities specifically the HZMT officers; - To collect actively information from the population mainly during home visits and to make a summary in the report; - To provide some financial incentives to CHWs
Men and their groups’ representatives	
- To sensitize the population specifically men on health problems, in order to increase their knowledge, enabling them to express easily their concerns and to monitor health centre activities, in collaboration with community associations (and churches); - To use CHWs’ networks to report their concerns and complaints; - To increase the number of CHWs - To use local associations/groups for informing the population; - To organize periodically meetings with community leaders, notables, local associations’ representatives, HC members, CHWs and health providers, invited by the health committee to discuss health concerns; - To improve the health centre supervision by the HZMT.	- To maintain CHWs and to improve their interface activities. - CHWs collected actively during home visits information from the community and to report them to health providers/The information collected actively by CHWs during home visits to be reported to health providers.. - To set in place a committee to which the population could also report their complaints and concerns. This committee will be composed of some community members coming from villages and CHWs, chosen by the community. - To organize meetings between this committee and health providers quarterly and a general assembly during which health providers could respond to community concerns. - This organization has to be preceded by a sensitization of the community.
Women and their groups’ representatives	
- To organize periodic meetings putting together community members and health providers in order to allow the population to directly bring forward their grievances about health services to health providers; - To use CHWs for collecting population’s concerns - To invite women to participate in these meetings by CHWs through their associations/groups; - To bring forward complaints and concerns directly to the person in charge of health facilities;	- To use CHWs for reporting complaints and concerns about health services, for avoiding health providers’ reprisals; - To train CHWs to bring forward their concerns to health providers; - Health providers have to discuss concerns of the population as a team for improving health services provision.

The CHWs, representatives of the health sector and local authorities had knowledge of the current process in health services because they had already been working within the community. Their suggestions were mainly based on their experiences of the local health sector and the national health policy. For example, CHWs suggested that they be trained on the interface role and provided with funds to cover expenditures occurring during their activities such as transportation for improving their work within the community. Moreover, they suggested a periodic involvement of other community leaders in health committee meetings so as to build a coalition around the concerns of the community. Nearly all the suggestions by the representatives of the health sector were also proposed by other groups.

### Integration and prioritization phase

The 48 suggestions made during the consultation phase were inputs for the integration phase. Facilitated by the research team, twelve participants previously engaged in the process discussed suggestions from each of the different groups with the aim to reach consensus on what the most important suggestions were, by looking at similar proposals from the different groups. Thus they regrouped 48 suggestions into 11 categories and finally articulated them in 6 themes as intervention components for social accountability (See Additional file 2).

Table 3 provides a mapping of the 11 categories of suggestions among the different groups. It emerged from the integration meetings that in Muanda and Bolenge the most widely supported suggestions to improve social accountability were almost the same and included: the use of CHWs’ networks, capacity building of the community, coalition building around social accountability, improvement of the management of concerns of the community by health providers at facility level, and the involvement of the HZ management team in community participation.

**Table 3:**
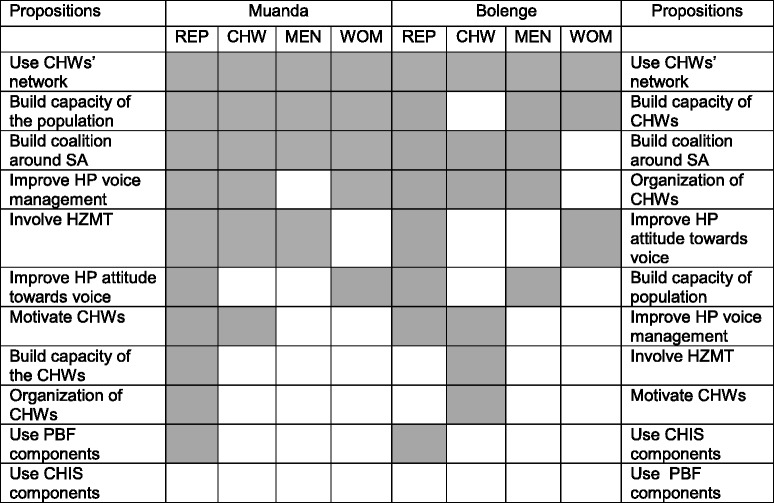
Mapping of Interventions proposition according to participant groups

*Legend:*

Gray coloured: mentioned by participant category

Number: Number of categories having mentioned the suggestion

*REP* representatives of health sector and local authorities, *CHW* Community health workers, *SA* social accountability, *HP* Health providers, *HZMT*, Health zone management team, *PBF* Performance based financing, *CHIS* Community health insurance scheme

The six themes that are proposed to be developed as intervention components to improve social accountability in maternal health services were formulated by clustering categories of suggestions having similar actions, those targeting the same actors or those that have to be implemented by similar actors: (1) Use CHWs and health committee(s) as interface for collecting community concerns; (2) Build the capacity of population especially women; (3) Build coalition around social accountability and maternal health through dialogue meetings; (4) Improve the involvement and support of the HZ management team to community participation; (5) Improve the attitude of health providers with regard to community concerns and the management of voice at health facility level, and; (6) Use of existing intervention integrating social accountability aspects.

### Programming phase

These six themes were presented and used as inputs by research partners during programming workshops. Research partners used suggestions coming from the integration meetings at the two sites. They agreed with the relevance of most of suggestions. They used them for drafting intervention components so as to adapt them to on-going interventions at the two research sites in order to increase community engagement (community, CHWs, health committee) and participation in social accountability mechanisms.

Below, we describe the emergent categories for which consensus was reached among all groups and that were validated after the 4 phases were finalised.

**Fig. 2:**
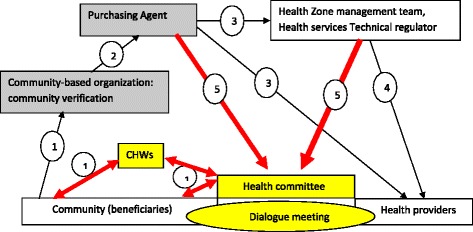
Cordaid’s Performances based Financing Model and modifications in Muanda. *Description:* In *yellow* and bold *red* are described the modifications introduced by community in the intervention carried out by Cordaid in Muanda. *Arrows* show how information is circulating in the model. Concerns from community previously collected through community verification (1) and transmitted to health zone management team (3) and to health providers (3) via the purchasing agent (2) are collected by community health workers (1) and transmitted to the health committee (1) in charge of organizing the dialogue meeting. The health committee will also receive information collected by community verification via the purchasing agent (5) and the HZMT (5). The health committee will send its feedback through CHWs, realizing the two-direction communication

**Fig. 3:**
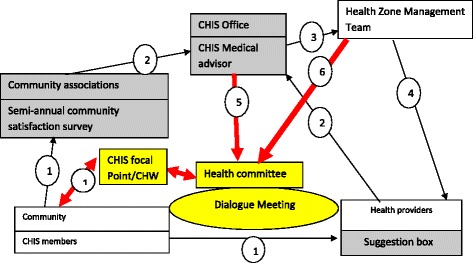
Medicus Mundi’s Community Health Insurance Model and modifications in Bolenge. *Description:* In *yellow* and bold *red* are described the modifications introduced by community in the intervention supported by Medicus Mundi in Bolenge. *Arrows* show how information is circulating in the model. Concerns from community previously collected through community survey and suggestion box (1) and transmitted to health zone management team (3) and to health providers (3) via the CHIS office (2) are collected by community health workers (1) and transmitted to the health committee (1) in charge of organizing the dialogue meeting. The health committee will also receive information collected by community survey via the CHIS office (5) and the HZMT (6). The health committee will send its feedback through CHWs, realizing a two-directional communication

1. Use community health workers and health committee as interface for collecting community concerns
  Participants from the different actor groups agreed that some community members work within the community as CHWs. They suggested that the latter might act as intermediaries between community members, especially women and health providers, for bringing forward their expectations, needs, complaints and questions. Participants observed that CHWs could bring up these needs and expectations at the health committee meeting, where any issues could be discussed and find ways to proceed, take appropriate actions and provide feedback. The mutual communication between CHWs and health committee members might facilitate a voice mechanism to bring community concerns to the health facility. The use of community health workers and health committee members as interface included (i) to collect the concerns of the population through the activities of community health workers, (ii) to build the capacity of CHWs and the health committee as interface between community and health providers, (iii) to organize activities of CHWs and the health committee, and (iv) to improve the motivation of CHWs. These social accountability activities are described below.
2. Collect the population’s concerns through the activities of community health workers
  All participants in the integration meetings in particular community members stated that most of the community members already recognised CHWs as people coming from their community; who passed by their homes or were in their community groups and who brought them information from the health facility. They also knew them as people who carried out health sensitizations and campaigns. Thus, they proposed that CHWs could also have a role in collecting information from the community during these activities. They asserted that the use of CHWs as intermediates could keep women from fear of reprisals as the women in the community trusted them, and would improve their involvement in voice and health services monitoring. Community members reiterated that they preferred to voice their concerns rather than switch to another health facility.
*“For avoiding conflicts, as we have CHWs, it is good that all the problems are declared to them and that when they come to the meetings, that they report them to the other members and health providers. It is known that the nurse in charge sits among them and when they will speak about it, the nurse in charge can listen to the complaints and the concerns of the community. He so can when he has meeting with his team at the level of the health centre, to talk about it to the other health providers to make adequate decisions”*
*(Community member, Woman, Bolenge)*
3. Build the capacity of community health workers and health committee as interface
  Most representatives of the health sector and local authorities and CHWs raised concerns about the lack of capacity of most CHWs in performing interface activities and proposed therefore to sensitize them, and to train them about their role as interface between health providers and the community. They proposed that the training should especially focus on the active collection of community information on the perception of health care provision but also on providing feedback. They thought that this training would improve the way CHWs worked within the community, improving their role as ‘bridge’. They asserted that CHWs needed to have skills and competences to carry out home visits, to provide feedback, and to manage confidentiality and anonymity of community members who came to them.
*“Thus it is necessary to focus on the training of CHWs, especially on the confidentiality. Because if CHW does not manage very well community concerns and discloses the identity of the community member who raised the concern, he will lose the confidence of the community”*
*(Representative, Woman, Bolenge)*
4. Organize activities of community health workers and health committees
  Most participants in the integration meetings noted that CHWs regularly visited homes and community groups. They proposed that during the home visits CHWs might collect data from community members, improve the reporting of mistreatments, follow-up with community members decisions made during health committee, and provide feedback to the community as well. However, they observed that CHWs did not have appropriate tools or resources for carrying out these activities.
  They therefore proposed to the HZ management team to provide them with appropriate tools and resources such as pens and notebooks for writing down community concerns. They also observed that CHWs and health committee (HC) members needed some resources, for instance funding of transportation costs for those coming from remote locations to the main village to attend meetings. They also proposed that CHWs and HC members required a notebook in which they could summarize their reports extensively during their meeting before discussing them at the health committee with health providers. Some participants, mostly CHWs, raised concerns about the key position of health providers in the health committee and their tendency to delete some information from the CHWs’ reports in final notes to be sent to the HZ management team. Participants also proposed to summarize decisions and actions proposed by the health committees with regard to community concerns, to bring them to the attention of community members using the CHWs’ network and to reach people who raised concerns.
*“The work of CHWs has to be formalized even by using a scrap of paper, it will allow a better follow of their activities”*
*(Representative, Man, Bolenge)*
5. Improve the motivation of community health workers
  Participants mostly CHWs, representatives of the health sector and local authorities raised the issue of motivation and the insufficient number of CHWs. They observed that currently active CHWs did not optimally cover all households in the health area for home visits, as per requirement of the national policy. According to them, this was the case because CHWs are not remunerated for activities they carry out within the community in constrictive socio-economic contexts. They proposed for instance to support some activities of CHWs by using performance based financing with indicators such as the number of households visits carried out, the number of patients brought to the health facility, and the number of concerns reported.
*“I would like to highlight and support this point, he raises something important, the heart of the problem. If there is funding or financial resources, I would suggest to provide us [CHWs] with a financial incentive…We will be more motivated to carry out community activities. We will work more efficiently”*
*(CHW, Man, Muanda)*
6. Build capacity of community members especially women
  Participants in both FGDs during consultation and the integration meetings recognized that one of the main reasons for women not voicing their concerns was their lack of knowledge/information on health service standards and what they were entitled to. They observed that women did not use opportunities such as CHWs’ and HCs’ network as a way of bringing forward their concerns. They then proposed to inform women through community sensitization, home visits, and health education sessions about health services standards and their entitlements, CHWs and HC. They proposed that CHWs could also bring information on community health best practices such as antenatal care, the immunization program, the importance of community voice in the improvement of the health services, and follow-up concerns as well. Participants also proposed to CHWs to use existing community groups and church as channels for reaching a larger audience.
*“It would maybe be better to speak about building their capacities. Maybe women in the community have some difficulties to express their complaints… But if we explain them the procedure to follow in case of an abuse in a health facility, if the woman understands that the fact that she raises her problem, brings forward her complaints, is in the way to improve, I believe that it is possible”*
*(Representative, Man, Bolenge)*
7. Build coalitions around social accountability through dialogue within the community
  From the suggestions by the FGDs, around approximately 50% of the participants in the integration meetings proposed to create a discussion platform beyond the HC in order to involve local leaders such as community groups’ representatives (women, men) and religious leaders. They argued that this discussion platform could counterbalance the power of the health providers, and their influences on the choice, decision-making and activities of CHWs and the HC. They proposed to include community leaders such as community groups’ representatives (women, men), village notables and administrative officers because of their influence in mobilizing people and diffusing information within their groups. Moreover, they included; men given their role in decision-making at household level, the current policy orientation emphasizing the involvement of men in maternal health and considering that women sometimes report to them some concerns about health services. Furthermore, religious leaders had to be included because of their current influence on their parishioners. The aim of these meetings, according to participants would be to increase the involvement and knowledge of other stakeholders on maternal health and social accountability, and to build a coalition around maternal health and social accountability in order to build a social pressure.
*“Could arrange it so that in the meetings of the health committee, even once a quarter, we invited even the village chiefs, the persons in charge of churches, secretaries of associations in case the president does not have time so that they come to hear what we discuss here? ”*
*(CHW, Woman, Muanda)*
8. Increase the involvement and the support of the health zone management team to community participation activities
  More than half of the representatives of the health sector and local authorities, and CHWs asserted that they had noticed that the HZ management team (HZMT) neglected community participation and did not appropriately support the activities of the CHWs. They stressed the importance of the HZMT in order to improve the organization of HC and the selection of CHWs. Representatives and CHWs required that the HZMT was involved in the training of CHWs and HC in their roles, and in the supervision of their activities in order to counterbalance the power of health providers as the HZMT could sanction the latter. They asserted that they would like to see the HZMT officer chairing the dialogue meeting, supervising personally the election of CHWs, and participating periodically in the HC meeting.
*“The Health zone management team chief officer also has to participate, personally, in some meetings of the HC, even in absence of the nurse in charge of the health centre, in order to learn himself about the community”*
*(Representative, Women, Muanda).*
9. Improve the attitude of health providers towards voice and the management of voice at health facility level
  According to almost all of the community members participating in the integration meetings, it could be the attitude of health providers towards voice and their position within social accountability mechanisms that were the main constraints for social accountability.
*“Because if CHWs do not manage to keep secret of the population and go so far as to say that such family told me that such nurse scolded them and if this one is not flexible to receive remarks, this will create a conflict. What will make that the user will be afraid to express his problem in these conditions…, we [health providers] must be flexible to receive remarks because if we ignite, it will be difficult to us to receive soon the complaints of the population and we shall not be capable of correcting our behaviour”.*
*(Representative, Man, Bolenge)*
  Almost all community members participating in the integration meeting proposed that the HZMT trains health providers on users’ voice, given their central position in social accountability in health services. This training would help to improve their attitude and disposition. Moreover, participants proposed that health providers possibly be trained on communication skills. Participants added that health providers are required to improve the health centre management in order to take into account population’s concerns, and regularly discuss them at the health centre as a team and to respond thereto. They also proposed that the local health centre put mechanisms in place for handling concerns and where it was unable do so refer these to the next level on the hierarchy. They thought that one step would be to manage and reduce the workload of health providers, to allow them to hear patients’ concerns, and to improve the working conditions of the former.
*“Health providers have to be sensitized because there are also badly educated health providers, who welcome badly patients or shout on them, who do not take into account their concerns or do not know how to manage patients’ needs”*
*(Representative, Man, Bolenge)*
10. Use existing intervention mechanisms
  Some participants especially representatives of the health sector and local authorities noticed that social accountability initiatives should not to be set up in isolation. They asserted that patients found it useful as a community to speak out as for groups using for instance community based health insurance. Representatives of the health sector from Muanda, proposed to use a community verification survey carried out by a community based organization for collecting community views about health services. They proposed also to set in place mechanisms for reporting results from the community verification survey to health providers and community members, and to improve the follow-up of recommendations, in order to make health providers more accountable (Fig. 2). Representatives of the health sector and local authorities from Bolenge proposed to use mechanisms set in place in community based health insurance such as to file concerns by means of a telephone or cellular call to the medical advisor, based on predominantly oral tradition culture and to complete to some extent the complaint books (Fig. 3).

### Adaptation and design

Based on what was suggested in the two health zones, research partners comprising of Cordaid representatives, Medicus Mundi, officers of the Ministry of Health and the research team adapted the health partners existing interventions in order to include the suggested components. It resulted in two variants depending on the existing interventions. Their goal for this adaptation was to increase community engagement and control of the social accountability mechanisms (community, CHWs, Health committee) rather than to have them in the control of the health zone level entities (HZMT, CHIS office, and the Purchasing Agent).

Research partners formulated an intervention proposal with three components: (1) Improving voice by creating a formal reporting system by using and improving community health workers and health committee’s activities; (2) improving answerability and responsiveness of health providers by enhancing the health committee and by training health providers on social accountability related aspects, and (3) Improving enforceability using social pressure by introducing a dialogue meeting and by involving the HZMT in their supervision.

The three components were proposed by research partners to be integrated as modifications in existing partners’ interventions or to be introduced even in the health area without existing interventions. They proposed also to introduce the three components in the selected health areas through a series of workshops carried out by the HZMT and described key intervention components, suggested implementation activities and underlined rationale (See Additional file 3).

## Discussion

While there is a growing interest in implementing social accountability mechanisms in health especially in maternal health services delivery, there are still limited insights into the involvement of beneficiaries in the design of suitable social accountability mechanisms and the type of mechanisms to use.

In this study, the first research question that needs to be answered is: Which social accountability mechanisms are needed in order to improve maternal health services responsiveness and performance? The use of DM allows participants to come up with a social accountability initiative that includes different components needed to be combined in order to address most of the challenges raised from the exploration and preparation phase [21]. The final selection of actions was based on the consensus among participants, reflecting inputs and perspectives of community members as well as of other participants involved. It includes three components: (i) Improving voice by creating a formal reporting system by using and improving community health workers and the health committee’s activities; (ii) improving answerability and responsiveness of health providers by enhancing the health committee and by training health providers on social accountability related aspects, and (iii) Improving enforceability using social pressure by introducing a dialogue meeting and by involving the HZMT in their supervision. Roughly, their proposal translates the improvement of the current community participation process in terms of improved organization and coordination of community activities having as expected outcome, an increased voice and fostered community enforceability. These outcomes are more likely to trigger the answerability of health providers.

The analysis of the proposed intervention shows that its components address at least the three core elements of the social accountability namely voice, enforceability and answerability [13, 24]. The proposed intervention makes allowance for the following to be fulfilled: “the premise that voice is ineffective unless it can elicit answerability and enforceability” [13, 25]. The proposed actions are consistent with literature on social accountability in the health sector [6, 14, 26–28] and community health workers [29–31]. For instance, the proposed components gather the two categories of factors, related to the health system and socio-cultural influences that according to Berlan and Shiffman [6] may shape health provider accountability.

In general, the intervention proposal leading to social accountability seems to be easy to translate into actions in order to improve social accountability in maternal health services, hence becoming feasible. Regarding support, all its components have been grounded on suggestions provided during the consultation phase and were assessed by health partners in accordance with the existing policy line. Secondly, their implementation in practice depends mainly on existing elements and resources such as community health workers, the health committee and health providers [32]. Furthermore, the proposed intervention by suggesting the link between CHWs and HC activities and the introduction of interface role increases the potential of improving community participation based social accountability, previously found to be ineffective when based on health committee only [6, 14, 28, 33]. In the suggested initiative, CHWs by actively collecting community concerns and communicating feedback from the health committee allow both to open and close the feedback loop [13, 34]. The transmission of community concerns to a strengthened health committee, anchored to other community stakeholders through the dialogue platform increases the potential of generating answerability of health providers. The link of the health committee, aware of its missions with local stakeholders increases its enforceability capacity [6, 35, 36]. The enforceability capacity in this model could also be increased by the involvement of the health zone management team, which possesses the supervision and control power on health providers.

The proposed model is mainly based on local elements, already existing at the local level in contrast with other social accountability mechanisms which use external actors for collecting community concerns and for exerting enforceability such as community score cards [37, 38] and performance based financing [28] or that promote inappropriate tools such as suggestion box in a context of high illiteracy [39]. However, the implementation of few of its activities need financial resources such as providing financial incentives to CHWs, notebooks for CHWs or financial support/remuneration for supervision. This could be a limitation, as their implementation is strongly dependent on the committement of health partners to financially support the programme due to government financial constraints and the constraining socio-economic context at the local level [40].

This study was also carried out to answer the second research question: How could community groups be involved in designing these social accountability mechanisms to make them more relevant? This study applied the DM in order to involve community participants in the intervention design process on social accountability. Previous studies which implemented the DM in the health sector applied it to involve participants; in a scientific advisory process to set a research agenda, in the development of clinical guidelines, and in the improvement of health research practices [18–20, 22, 41]. The evaluation of the DM applied to these research studies showed that the DM demonstrated the effective participation of stakeholders involved and allowed to usefully and adequately reflect the perspectives of participants [18–20, 22]. We consider that our study successfully implemented the DM as it followed the process as described in seminal papers [18]. Moreover, our study implemented the phases from: consultation to programming, formally set as part of this research and the resulting intervention proposal reflects participants’ perspectives taking into account local contexts [20]. Furthermore, in our study, community members were facilitated to develop their own voice and suggestions, and they were prepared for integration with other stakeholder groups namely health providers and local authorities. Participants were able to explain and justify their propositions. This provides a participatory process similar to those provided by other participative approaches such as the community-based intervention using local facilitators, co-creation and co-creating knowledge translation [42–44].

In this study, we implemented most of the key elements which were described as the strengths of the DM [18] i.e. to acknowledge and facilitate different groups of stakeholders’ influence on the intervention design, and to guard procedural fairness. In our study, different participant groups were able to participate in the process as we used non-technical language and scientific knowledge was not presupposed. They provided their suggestions based on their values, experiences and knowledge. Furthermore, the process provided opportunities for knowledge sharing between participants and mutual learning. Its interactive character stimulated co-construction of the suggested intervention components [32]. For example, CHWs suggested to be paid or to receive other forms of motivation, while health providers and managers who are supposed to apply the national health policy considered CHWs as volunteers. Community members expressed their concerns about the attitude of health providers regarding the community voice as they anticipated possible responses from the health providers, and CHWs made others aware that they did not have notebooks and pens for documenting their activities.

We observed that the strategy to let different groups meet separately prior to the integration meetings stimulated an open-exchange of experiences among equals allowing each group to build its own point of view. The integration meetings provided opportunities to representatives of different groups to sit together and to build by consensus a shared proposal. Furthermore, the researcher as facilitator took care that in the discussion all groups were represented in a balanced way, this was supposed to prevent community members from being overruled by other groups such as health providers and health managers, even though, it is known that the balance of numbers does not necessarily equal the balance of power [20, 32]. This facilitation process was handled by the research team, independent from health service providers, community groups and NGO partners. All participants were equally treated and discussions were open during all meetings, as well as respectful and collaborative in the integration meeting.

### Implication for policy and practice

The proposed intervention as described in the present study suggests some modifications in the national health policy with regards to community participation and improvements in health system practices [28, 45]. Findings of this study suggest to clearly insert in the missions of CHWs, the active collection and the transmission of community concerns [34]; and in the missions of HC, the interface role, the management of community concerns collected through CHWs and the organization of dialogue meetings. In terms of practices in the health system, the findings of this study suggest an improvement in the organization and the operation of community participation, and an improvement of its supervision by the health zone management team [34, 46, 47]. The study findings also raise some issues such as the motivation of CHWs and the central position of health providers in community participation [34, 47, 48],

### Study limitations

This study had some limitations related to the study design as the DM is based on focus group techniques. For instance, the DM itself, was applied in a context characterized by asymmetry of knowledge and powers [20, 32]. This situation presents a risk for one group to be dominated by another, thereby losing its knowledge inputs. Despite the adoption of a method designed to minimize an unequal power dynamic and asymmetries between participants, there is an inherent inequity between community members and representatives of health sectors and local authorities. This was an on-going ethical concern for us. However by using the DM process, we had tried to be attentive to preventing asymmetries and creating a fair and meaningful process. Some precautions were set: the separation of stakeholder groups in the first round of focus groups, an equal number of participants for each of the groups in the dialogue meetings, the selection of open-minded participants, the use of non-technical language, the equal distribution of speaking time, the respect of conversation time, the assistance of community groups and a fair facilitation being transparent and equitable in our partnership with participants. Additionally, the DM as strength, the management of the meeting enabled a dialogical process rather than a shifting of control process. According to Abma and Broerse [20] the integration meeting stimulates mutual learning between stakeholders by the development of a shared action proposal supported by all participants, as they spend adequate time to build reciprocal relationships and to foster mutual respect and knowledge integration. A further strength of the DM is its use of the different phases in the dialogue process for building consensus and enabling the different perspectives to be included despite the asymmetry of knowledge and power.

A second limitation relating to the DM is the representativeness of the participants and the actions identified. In the organization of the process, we used purposive sampling as we preferred to find community members committed to improving maternal health service, and possibly committing themselves to actions and follow-up implementation. Even though criteria were used, their implementation by the research team could have been biased due to the researchers’ subjectivity and this may unintentionally have led to the exclusion of the most marginalised and vulnerable participants. However, at the same time, we verified our previous focus group discussions as to whether these community members were still in line with the rest of the community. Furthermore, the integration meeting provided us with the participants’ insights in the support of the various suggestions that were collected and that were made by participants. Finally, the credibility of our findings has been enhanced through validation by participants. The fairness was warranted through the open and respectful participation and the consideration of their inputs in the final proposal [20, 32].

Thirdly, the study was neither designed to be nationally representative in the action proposed, nor representative of a particular health zone. However, the distinct characteristics of the two communities enabled us to generalize the robustness and potential of the proposed intervention to raise social accountability in maternal health services as well as in all local health centres that provide maternal health services as part of a comprehensive healthcare package.

### Research team and reflexivity

As with any qualitative content analysis, interpretation could be influenced by the background and views of the research team members. Thus, in this study, even though the data collection and analysis were mainly performed by the first author, findings were discussed with supervisors, local health partners and community members, to support trustworthiness.

## Conclusion

The use of the Dialogue Model facilitated the involvement of community beneficiaries amongst women with other stakeholders having different perspectives and types of knowledge in a participatory advisory process and to articulate their suggestions on a combination of social accountability intervention components, which address the three core elements that need to be minimally present, voice, answerability and enforceability. Even though this intervention proposal is specific for the two health zones contexts, it is practically feasible. Its components drawn upon suggestions coming from the stakeholders are mostly in line with the current health policy and could be easily implemented as they used existing resources but need additional (financial) resources only for supervision and support.

## Additional files

Additional file 1: Summary of the situation analysis of social accountability mechanisms in rural setting in the DRC. (DOCX 27 kb)

Additional file 2: Coding matrix. (XLSX 13 kb)

Additional file 3: Key intervention components as formulated by research partners during programming phase. (DOCX 17 kb)

## Abbreviations

CHIS: Community based health insurance scheme
CHWs: Community Health Workers
DM: Dialogue Model
DRC: Democratic Republic of the Congo
FGDs: Focus group discussion
HC: Health committee
HZ: Health Zone
HZTM: Health zone management team
NGO: Nongovernmental organizations
