# Supplementary material for: Participatory approach to design social accountability interventions to improve maternal health services: a case study from the Democratic Republic of the Congo
Source: Glob Health Res Policy. 2017 Feb 6;2:4. doi: 10.1186/s41256-017-0024-0 (PMC5683322; doi:10.1186/s41256-017-0024-0)
Supplement: Supplementary file 1 — Summary of the situation analysis of social accountability mechanisms in rural setting in the DRC. (DOCX 27 kb) [file 41256_2017_24_MOESM1_ESM.docx]

**Appendix 1**. **Summary of the situation analysis of social accountability mechanisms in rural setting in the DRC (Mafuta *et al*, 2015)**

In a previous study exploring existing social accountability mechanisms in rural settings in the DRC, we found that women were positive regarding the health care that they received and very few were able to express clearly during interviews their concerns about health care or health providers. We also found very few women voiced their concerns and complaints about health services to health providers. Interviews revealed that women in these settings were not used to expressing their concerns and did not develop this habit. Therefore, we noticed that women raised very few expectations in order to improve the health service provision.

In their expectations, they only emphasized health service inputs such as assigning a doctor in the local health centre, extending the health service centre with more wards, supplying drugs and equipment and providing free care. They did not emphasize the quality of care or health providers’ behaviour.

In addition, we discovered that women did not know either how to transmit their concerns to relevant actors and decision makers or how their concerns were managed within the health services. Among reasons that could explain this situation were the absence of procedures to express them, the lack of knowledge thereof, the fear of reprisals, of being misunderstood as well as factors such as age-related power, ethnicity backgrounds, and women’s status.
