# Supplementary material for: Participatory approach to design social accountability interventions to improve maternal health services: a case study from the Democratic Republic of the Congo
Source: Glob Health Res Policy. 2017 Feb 6;2:4. doi: 10.1186/s41256-017-0024-0 (PMC5683322; doi:10.1186/s41256-017-0024-0)
Supplement: Supplementary file 3 — Key intervention components as formulated by research partners during programming phase. (DOCX 17 kb) [file 41256_2017_24_MOESM3_ESM.docx]

Table 5. Key intervention components as formulated by research partners during programming phase

| Key interventions components | Implementation activities | Rationale |
| --- | --- | --- |
| (1) Create a formal reporting system for community concerns (Voice) | - set community health workers and health committee as a formal systems for collecting, reporting and handling community concerns | - Strengthen an existing community-health providers interface systems. |
| (1.1) Improve home visits of community health workers | **Health zone management team, health providers and community level**   - train CHWs on social accountability and interface role, specifically mechanisms and methods to encourage community members to report concerns; - improve the content of existing national policy guidelines specifically the home visit guidelines by including among activities to carry out by CHWs the collection of community concerns about health services, the report of health services performances and health committee feedback; - add among information to provide to community members during home visits information on CHWs, Health Committee and their link with the health centre, social accountability concepts; - provide CHWs with notebooks and pens for documenting their duty; - strengthen the network CHWs and HC, - train CHWs to send to Health committee a summary as brief monthly anonymous updates including concerns articulated by community member; - involve the HZMT officer in charge of community activities in direct supervision of CHWs and HCs activities in quarterly basis and in their training; - provide CHWs with small incentives and motivation under performance-based financing scheme to fulfil some specific targeted activities related to their role of interface | - CHWs are volunteers who use a part of their time performing community-based health activities. They accomplish their duty mainly through home visits. Every CHW is in charge of visiting 15-25 households every month and has to report through their representatives to the health committee; - The integration aimed to use home visits for collecting concerns, questions and expectations of community members. It also aimed to use home visits for informing community members about health services issues in order to build their capacity in terms of knowledge and information, as well for bringing feedback from the health committee about concerns raised during home visits; - The improvement of home visits will improve community engagement and community oversight as CHWs are members of the community and women manifest more confident and are trustful to them. - CHWs could carry out home visits without financial interest and with lower risk of friction and pitfall with health providers as home visits are their usual duty. - This documentation of CHWs activities could be used to assess CHWs’ activities, essential basis for introducing a performance based financing system for motivating them |
| (1.2) Improve activities of the health committee by introducing the management of population concerns and steering of dialogue meetings. | **Health zone management team and community levels**   - set the health committee at local level as the entity that manages population‘s concerns and complaints and steers dialogue meetings; - propose to partners such as Cordaid PBF and CBHIS to summarize and send to the Health Committee, in quarterly frequency through the HZTM information they gathered through the respective systems; - train the health committee members to summarize data from CHWs, to discuss them with the health providers during meetings and to send them to HZMT; - train health committee members on advocacy; - train health committee members on steering the dialogue meeting. | - The component aimed to shift down the management of population’s feedback from Health zone level to the local level in order to increase providers’ responsiveness to the community; - Information coming from partners added that was collected by CHWs is supposed to increase the involvement of the population through the health community in the monitoring of health services; - The advocacy skills could be used for improving support from the HZMT to take into account their report and to use it during the PHC Monitoring meeting. The latter is very important in order to set up a social accountability mechanism at the health zone level especially for the General referral Hospital that has currently not an interface system. - The training could specifically emphasize how to identify and contact community leaders, how to prepare the dialogue meeting, how to use tools and information process for summarizing relevant data, how to involve them in their meetings, how to conduct this meeting taking into account their perspectives. |
| (2) improve enforceability and answerability using social pressure by introducing a dialogue meetings | **Community level**   - set the organization of a dialogue meeting as a mandatory activity for the heath committee, under the supervision of the HZMT; - use the dialogue meeting as the space where the health committee invites and interacts with other community leaders (social pressure), including local authorities, representatives of community groups, present information gathered through voice mechanisms, discuss them with the health providers (Answerability) and take action; - organize prior to the dialogue meeting an internal dialogue between health providers and the health committee for discussing draft reporting issues and for collecting/soliciting actions for addressing the raised issues; - use dialogue meetings to evaluate actions set in previous meetings; - Encourage the health centre to fund the dialogue meeting as a health committee meeting expanded to other stakeholders; - publish a summary of the dialogue meeting on a poster in the local health centre or in local community groups’ offices. | - Dialogue meetings were proposed to be organized every three or six months in order to enhance the coalition around health activities, social accountability, citizen monitoring of health services, and dialogue between the community and the health providers; - The internal dialogue meeting aimed to build a learning cycle to deal with concerns and complaints in a constructive way, to make the process less aggressive and to enhance compliance of health providers, - The health committee present during dialogue meeting real concerns of the community and the health providers’ actions; - The publication of dialogue meeting minutes allows the population to link their complaints, suggestions and ideas to perceived change made in the health services by health providers. |
| 1. Improve the answerability and responsiveness of health providers | **Health zone management team level**   - Carry out through the HZMT a training workshop and supportive supervision with health providers in order to encourage them to be receptive to concerns raised by the community, to discuss the raised concerns as a team in the health centre, and to establish an action plan translating their engagement and commitment to be more responsive to raised concerns. - Work with health providers in order to improve the monitoring of health care service by the population through health committee; - Conduct counselling for health providers to set up systems for collecting patients’ views as customers (suggestion box, filling forms, face-to-face interviews, official meeting with the community leaders, and informal discussion with customers or community, direct feedback of customers to HP) and to provide feedback to community.   **Health providers level**   - Participate in the dialogue meeting, - Define a set of health centre performance indicators to provide to the health committee as information for the community during the health committee meeting in order to improve community awareness of the health service. | - The component addresses concerns raised by the participants of the focus groups and dialogues about the attitude of health providers towards voice and their central position in the social accountability process, - The component aimed to the improvement of the quality of the response of health providers to community members’ concerns - HZMT officers are concerned as direct supervisors and superior of health providers at local level; - The publication and the dissemination of health centre’s data and statistics aims at improving community awareness of the health service. |
